# Clinical efficacy of donepezil combined with nerve growth factor in the treatment of patients with Parkinson’s disease dementia and its impact on adiponectin and soluble tumor necrosis factor-alpha receptor-1

**DOI:** 10.12669/pjms.41.2.11191

**Published:** 2025-02

**Authors:** Meiling Hu, Jiamei Ye

**Affiliations:** 1 Meiling Hu, Department of Neurology, Taizhou First People’s Hospital, Taizhou, Zhejiang Province 318020, P.R. China; 2 Jiamei Ye, Department of Neurology, Taizhou First People’s Hospital, Taizhou, Zhejiang Province 318020, P.R. China

**Keywords:** Adiponectin, Donepezil, Nerve growth factor, Parkinson’s disease dementia, Soluble tumor necrosis factor receptor-1

## Abstract

**Objective:**

To explore clinical efficacy of donepezil combined with nerve growth factor (NGF) in the treatment of Parkinson’s disease dementia (PDD) and its potential impact on serum levels of adiponectin (APN) and soluble tumor necrosis factor receptor-1 (sTNFR-1).

**Methods:**

Clinical data of 140 patients with PDD treated in Taizhou First People’s Hospital from March 2021 to December 2023 were retrospectively analyzed. Patients were grouped based on the treatment received. Patients who received donepezil alone (n=68) comprised the Donepezil group, and patients who were treated with a combination of donepezil and NGF (n=72) were assigned into the Donepezil & NGF group. Treatment effects, symptom improvement before and after the treatment, APN and sTNFR-1 levels, and incidence of adverse reactions were compared between two groups.

**Results:**

The overall efficacy of the combination therapy was higher than that of donepezil regimen alone (*P*<0.05). After treatment, symptom improvement in the Donepezil & NGF group was significantly better than that in the Donepezil group (*P*<0.05). Post-treatment serum levels of APN in the Donepezil & NGF group were significantly higher than that of Donepezil group, while the level of sTNFR-1 was significantly lower (*P*<0.05). There was no significant difference in the incidence of adverse reactions between the two groups (*P*>0.05).

**Conclusion:**

Combined regimen of donepezil and NGF is more effective than donepezil monotherapy in improving cognitive function, neurological function, and severity of the condition of patients with PDD, and is associated with the suppression of the inflammatory response without a significant increase in the incidence of adverse reactions. The study provides a basis for the clinical application of the combined regimen, but it still needs to be confirmed by more basic and clinical research.

## INTRODUCTION

Parkinson’s disease (PD) is the second most common neurodegenerative disease of older populations.^1^ Cognitive impairment is common in PD and about 30-35% patients with PD suffering from dementia, a rate that is 4-6 times higher than the general population.^2^ Parkinson’s disease dementia (PDD) is characterized by deficits executive and visuospatial functioning, as well as impaired cognition and memory.^3^ PDD requires ongoing treatment, and the cost of care and medical treatment can impose a heavy social and economic burden.^4^,^5^ PDD, thus, not only significantly impacts physical and mental health of patients, but is also associated with a heavy socio-economic and medical burden.

Research shows an important role of inflammatory factors, such as adiponectin (APN) and soluble tumor necrosis factor receptor-1 (sTNFR-1), in neuronal damage and apoptosis that are linked to neurodegenerative manifestations in patients.^6^,^7^ APN belongs to endogenous bioactive peptides family, and plays a role in regulating insulin sensitivity. Current studies have showed that APN has anti-atherosclerosis and anti-inflammation functions, and its expression levels are closely related to non-motor symptoms in patients with PDD,^8^,^9^ while sTNFR-1, a TNF-family receptor, can activate signal transduction and mediate inflammatory responses in neurons. Therefore, it is believed that evaluating the changes in serum expression of sTNFR-1 and APN can help clarify the therapeutic effect of the disease.

Donepezil, a reversible central acetylcholinesterase inhibitor, is routinely used in the clinical treatment of PDD. Donepezil inhibits central acetylcholinesterase activity, slows down acetylcholine breakdown, and increases acetylcholine content, thus improving cognitive function and alleviating clinical symptoms of PDD.^10^,^11^ However, studies show that the efficiency of donepezil monotherapy is limited, and long-term use may cause related adverse reactions.^10^-^12^ Nerve growth factor (NGF) is commonly used in clinical treatment of neurological diseases due to its ability to promote neuronal growth and development of central and peripheral neurons, inhibit neuronal damage and maintain the function of the nervous system.^13^

Currently, studies investigating the efficiency of combined donepezil and NGF therapy in treating PDD symptoms are scarce. A study by Liu et al.^14^ showed that NGF combined with donepezil improves the clinical efficacy of patients with PD. However, the impact of the combined regimen on the serum expression of sTNFR-1 and APN is still unclear. The aim of this retrospective study was to explore the clinical efficacy of donepezil combined with NGF in the treatment of PDD.

## METHODS

Clinical data from 140 patients with PDD treated in Taizhou First People’s Hospital from March 2021 to December 2023 were retrospectively selected. Patients were grouped based on the treatment received: Donepezil group (n=68, donepezil alone) and Donepezil & NGF group (n=72, a combination of donepezil and NGF).

### Ethical approval:

This retrospective study was approved by the hospital ethics committee with the number: 2024TR022, Date: March 18^th^ 2024.

### Inclusion criteria:

- Patients met the diagnostic criteria for PDD.^15^
- Patients with Hoehn and Yahr (H-Y) staging at level I-III.
- Patients received treatment with donepezil or donepezil combined with NGF.
- Patients with complete clinical data.

### Exclusion criteria:

- Patients with other neurological disorders.
- Patients with secondary PDD caused by vascular diseases.
- Patients with renal and liver dysfunction.
- Patients with brain tumors.

### Treatment:

### Basic treatment:

Both groups were treated with basic treatment. Pramipexole (Shiyao Group Ouyi Pharmaceutical Co., Ltd., specification: 0.25mg *30 tablets) was administered orally as 0.125 mg/time, three times/day for the first week; 0.250/time, three times a day for the second week; and then increased by 0.750 mg at intervals of one week, with a maximum dose of 4.5mg per day.

### Donepezil group:

Patients in the Donepezil group also orally took Donepezil (Shaanxi Ark Pharmaceutical Co., Ltd., specification: 5mg * seven tablets) 5mg/time, once a day.

### Donepezil & NGF group:

On the medication of Donepezil, patients in the Donepezil & NGF group were also given Mouse NGF for Injection (Lizhu Pharmaceutical Factory of Lizhu Group, specification: 30 μg/bottle), intramuscular injection of 30 ug/time, once a day.

Patients in both groups were treated for three months.

The observation indicators were as follows:

### Treatment effect:

According to the Unified Parkinson’s Disease Rating Scale (UPDRS)^16^, a decrease of ≥ 65% in UPDRS was considered significant; 30% to 64% reduction in UPDRS was effective; A reduction of less than 30% in UPDRS was considered.

### Symptom improvement:

- Cognitive function was evaluated using the Mini Mental State Examination (MMSE)^17^, with a total score of 30 points, and a higher score indicated better cognitive function.
- Neurological function was evaluated using the Scale for Outcomes in Parkinson’s Disease for Autonomic Symptoms (SCOPA-AUT)^18^, which ranges from 0 to 69 points, with lower score indicating better neurological function.
- Severity of the condition was assessed by the UPDRS, which ranges from 0-147 points, with higher scores indicating more severe symptoms.
- APN and sTNFR-1 levels were measured in the fasting blood serum of patients using enzyme-linked immunosorbent assay.
- Adverse reactions including drowsiness, anorexia, dry mouth, and abdominal discomfort.

### Statistical analysis:

SPSS version 25.0 (IBM Corp, Armonk, NY, USA) was used for data analysis. For continuous variables, data were presented as mean and standard deviation (SD). Paired t-tests were used to determine differences within groups, while independent sample t-tests were used for inter group comparisons at each time interval. The hypothesis of equal variance was examined and considered in the analysis. For categorical variables, frequency distribution was provided and expressed as a percentage. The chi square test was used to compare the categorical variables between the two groups, such as gender distribution and H-Y grading. A p-value less than 0.05 was considered statistically significant. All reported p-values were bilateral. PRISM8.0 software (GraphPad, San Diego, USA) was used for graphical presentation of the results.

## RESULTS

**Fig.1:**
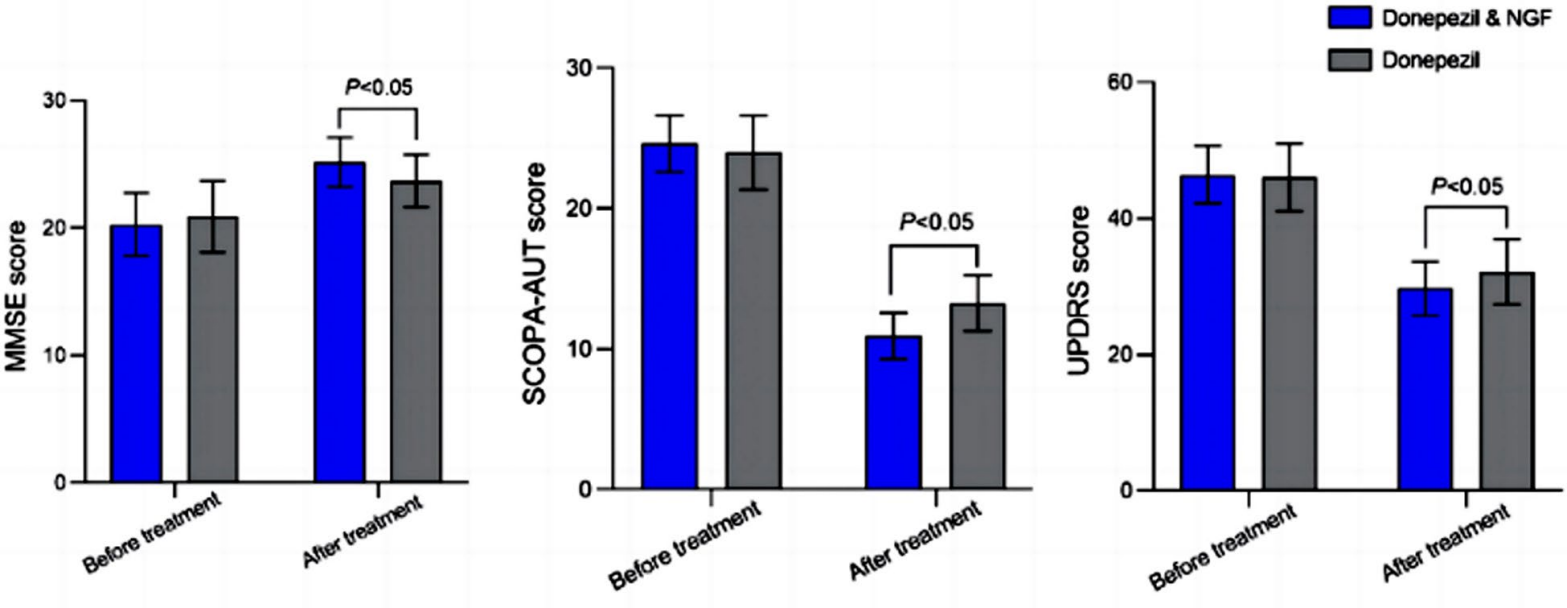
Comparison of symptom improvement before and after treatment between two groups. NGF: nerve growth factor; MMSE: Mini Mental State Examination; UPDRS: Unified Parkinson’s Disease Rating Scale; SCOPA-AUT: autonomic symptoms.

Clinical data of 140 patients (84 males and 56 females) were included in this retrospective study. Age of the patients ranged from 43 to 78 years, with an average age of 60.33 ± 6.92 years. Based on the treatment, 72 patients who received the combined regimen were included in the Donepezil & NGF group and 68 patients on donepezil monotherapy comprised the Donepezil group. There was no significant difference in baseline data between the two groups (*P*>0.05) (Table-I). The overall efficacy rate of the Donepezil & NGF group was significantly higher than that of the Donepezil group (*P*<0.05) (Table-II). There was no significant difference in the MMSE, SCOPA-AUT, and UPDRS scores between the two groups before the treatment (*P*>0.05) (Fig-1). After the treatment, MMSE scores of the two groups significantly increased, while SCOPA-AUT and UPDRS scores significantly decreased compared to pre-intervention.

**Table-I:** Comparison of baseline characteristics between two groups.

Characteristics	Donepezil & NGF (n=72)	Donepezil (n=68)	t/χ^2^	P
Male (Yes, [n(%)])	41 (56.94)	43 (63.24)	0.577	0.448
Age (years, mean ± SD)	61.07±7.21	59.54±6.56	1.307	0.193
Disease course (year, mean ± SD)	4.19±1.48	4.46±1.62	-0.997	0.321
***Educational level [n(%)]***				
Junior high school and below	44 (61.11)	39 (57.35)	0.205	0.651
High school and above	28 (38.89)	29 (42.65)		
***H-Y staging [n(%)]***				
Level I	17 (23.61)	12 (17.65)	2.239	0.326
Level II	48 (66.67)	44 (64.70)		
Level III	7 (9.72)	12 (17.65)		
Hypertension (Yes, [n(%)])	27 (37.50)	20 (29.41)	1.026	0.311
Diabetes (Yes, [n(%)])	14 (19.44)	16 (23.53)	0.347	0.556
Coronary heart disease (yes, [n(%)])	19 (26.39)	15 (22.06)	0.357	0.550

H-Y staging, Hoehn and Yahr Staging.

**Table-II:** Comparison of treatment effects between two groups [n(%)].

Group	n	Significant effect	effective	Invalid	Overall effective rate
Donepezil & NGF	72	37 (51.39)	32 (44.44)	3 (4.17)	69 (95.83)
Donepezil	68	27 (39.71)	31 (45.59)	10 (14.71)	58 (85.29)
*χ^2^*					4.612
*P*					0.032

**Fig.2:**
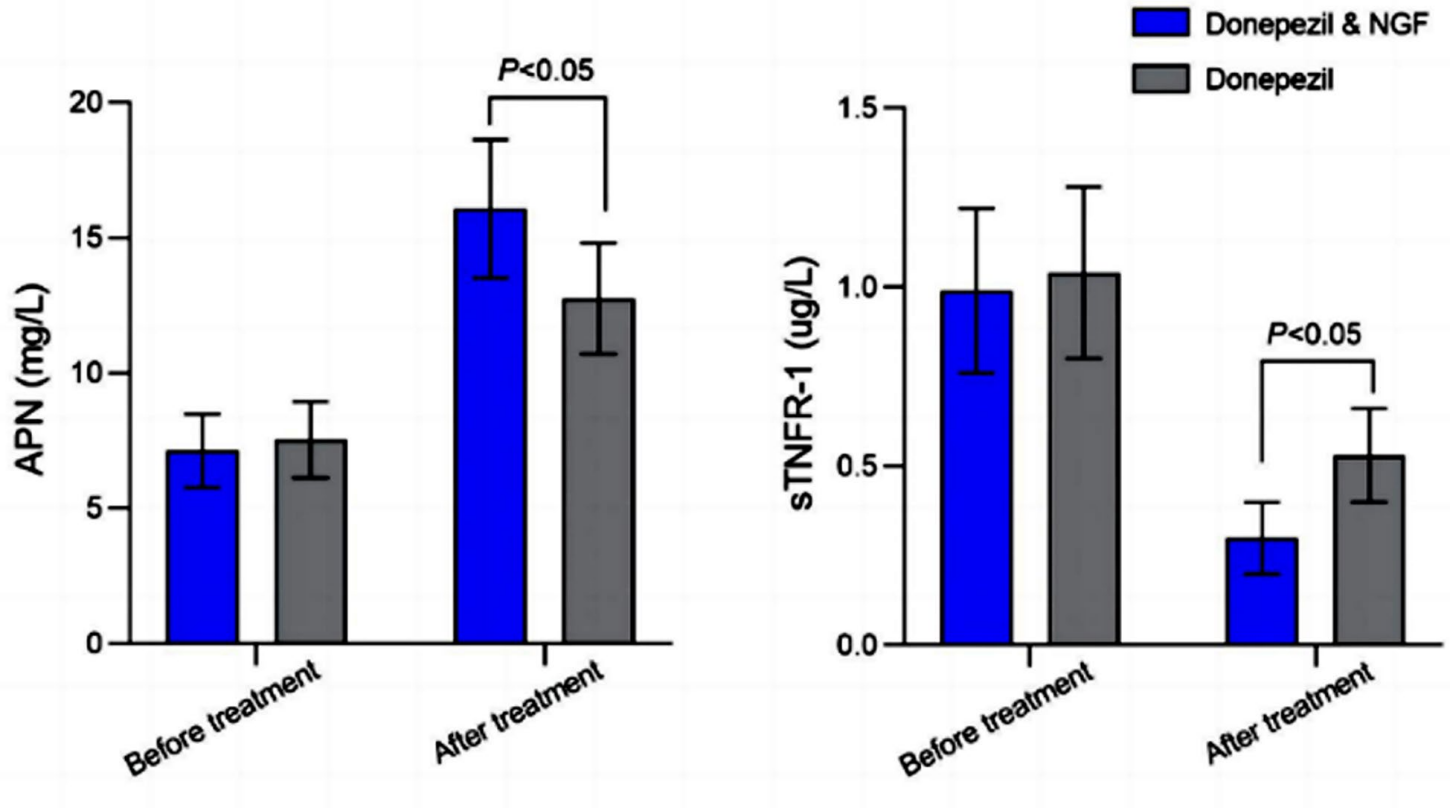
Comparison of serum indicator levels before and after treatment between two groups. NGF: Nerve growth factor; APN: Adiponectin; sTNFR-1: soluble tumor necrosis factor receptor-1.

Post-intervention MMSE score of patients who received the combined medication regimen was significantly higher, and SCOPA-AUT and UPDRS scores were significantly lower than those of patients on donepezil alone (*P*<0.05). Serum levels of APN and sTNFR-1 were comparable in the two groups before the treatment (Fig.2; *P*>0.05). After the treatment, serum APN levels in both groups increased, and sTNFR-1 decreased compared to pre-treatment values. Patients, treated with a combination of donepezil and NGF had significantly higher post-intervention levels of APN and lower levels of sTNFR-1 compared to patients on donepezil monotherapy (*P*<0.05). There was no significant difference in the incidence of adverse reactions between the two groups (*P*>0.05) (Table-III).

**Table-III:** Comparison of incidence rates of adverse reactions between two groups [n(%)].

Group	n	Drowsiness	Anorexia	Dry mouth	Abdominal discomfort	Overall incidence rate
Donepezil & NGF	72	3 (4.17)	2 (2.78)	4 (13.89)	1 (1.39)	10 (13.89)
Donepezil	68	2 (2.94)	1 (1.47)	3 (4.41)	2 (2.94)	8 (11.76)
*χ^2^*						0.141
*P*						0.707

## DISCUSSION

The results of this study showed that combined donepezil and NGF regimen leads to more significant clinical effect in patients with PDD than monotherapy with donepezil. Compared to donepezil alone, combined treatment is equally safe and is associated with more stable levels of APN and sTNFR-1. Our results further confirm that the combination of donepezil and NGF is effective in treating PDD, can improve cognitive function, alleviate the severity of the condition, and enhance treatment efficacy without increasing the risk of adverse reactions. At present, acetylcholinesterase inhibitors, such as donepezil, are most commonly used for treating PDD.^19^,^20^

However, a study by Baik K et al.^21^ showed that while donepezil was able to effectively regulate EEG of patients with PDD, the improvement in cognitive function was not significant. Sawada H et al.^22^ confirmed that donepezil can improve cognitive function in patients with PDD to a certain extent but has no preventive effect on their mental symptoms. The above studies indicate that acetylcholinesterase inhibitors alone are insufficient to achieve clinical expectations in the treatment of PDD.^21^,^22^ Studies have demonstrated the effectiveness of NGF in promoting the development of peripheral and central neurons, and inhibiting sustained neuronal damage in patients with PD.^13^,^14^ Liu Y et al.^14^ found that NGF in combination with the conventional pharmaceutical treatment regimen was able to increase the total efficacy of PD treatment to 88.46%, significantly improving neurological function of the patients. A study by Tome D et al.^23^ confirmed that NGF can promote local damaged nerve repair and enhance brain injury tolerance. It is plausible that NGF enhances cognitive function and treatment outcomes in patients with PD by establishing neural pathways, repairing hippocampal neurons, and reconstructing damaged neural function.

Our study also found that the MMSE score of patients treated with NGF significantly increased. The main component of NGF extracted from the submandibular gland of mice. Studies showed that mouse NGF can prevent sustained neuronal damage by inhibiting the release of toxic amino acids, regulating cholinergic activity, inhibiting neuronal apoptosis, and other pathways.^13^,^14^,^19^,^20^,^24^ Chen X et al.^25^ demonstrated that the combination of mouse NGF and donepezil exerted therapeutic effects by inhibiting certain inflammatory cytokines in elderly dementia patients, alleviating dementia symptoms and improving cognitive function. Our results support the above research viewpoint by comparing clinical efficacy and adverse reactions. We showed that a combination of NGF and donepezil is beneficial for the treatment of PDD, can reduce neuronal damage, significantly improve therapeutic effect, and is safe.^14^,^25^

The results of this study demonstrated combined regimen led to higher post-treatment levels of APN and lower levels of sTNFR-1 compared to donepezil monotherapy. We may speculate that both drugs have synergistic effect in patients with PDD. Since PD is also a neuroinflammatory disease,^26^ donepezil can successfully inhibit oxidative stress response, reduce generation of reactive oxygen species, and alleviate inflammatory symptoms.^21^,^22^,^27^ At the same time, NGF antagonizes excitatory amino acid toxicity, inhibits local neuroinflammatory response, and prevents secondary brain injury after acute cerebral hemorrhage.^27^-^30^ Our study further confirms the feasibility and effectiveness of adding NGF to the routine treatment of patients with PDD.

### Limitations:

In addition to symptom improvement, the study also confirmed the effect of the combination of donepezil and NGF on APN and sTNFR-1. However, the study also has some limitations. Firstly, this is a single-center small-sample size retrospective study which may limit the generalizability of the results. Secondly, further follow-up analysis is needed to confirm the impact of NGF combined with donepezil on long-term functional recovery in patients. Thirdly, further basic and clinical research is needed to investigate pathways and mechanisms through which NGF and donepezil exert their therapeutic effect in patients with PDD.

## CONCLUSION

Compared with donepezil monotherapy, a combination of donepezil and NGF can more effectively improve cognitive function, neurological function, and severity of the condition of patients with PDD. Combined treatment regimen enhances treatment efficacy, and regulates APN and sTNFR-1 levels without a significant increase in the incidence of adverse reactions. The study provides a basis for the clinical application of the combined donepezil and NGF, but it still needs to be confirmed by more basic and clinical research.

### Authors’ Contributions:

**MH:** contributed to the study design and manuscript writing.

**MH** and **JY:** data collection, data analysis and interpretation.

**JY:** Was involved in the manuscript revision and validation and is responsible for the integrity of the study.

All authors have read and approved the final manuscript
